# The Effect of Lipoic Acid on Cyanate Toxicity in Different Structures of the Rat Brain

**DOI:** 10.1007/s12640-013-9395-2

**Published:** 2013-04-27

**Authors:** Maria Sokołowska, Elżbieta Lorenc-Koci, Anna Bilska, Małgorzata Iciek

**Affiliations:** 1 The Chair of Medical Biochemistry, Jagiellonian University Medical College, 7, Kopernik Street, 31-034 Kraków, Poland; 2 Department of Neuropsychopharmacology, Institute of Pharmacology, Polish Academy of Science, 12, Smętna Street, 31-343 Kraków, Poland

**Keywords:** Cyanate, Lipoate, Sulfane sulfur, Hydrogen sulfide, Atherosclerosis

## Abstract

Cyanate is formed mostly during nonenzymatic urea biodegradation. Its active form isocyanate reacts with protein –NH_2_ and –SH groups, which changes their structure and function. The present studies aimed to investigate the effect of cyanate on activity of the enzymes, which possess –SH groups in the active centers and are implicated in anaerobic cysteine transformation and cyanide detoxification, as well as on glutathione level and peroxidative processes in different brain structures of the rat: cortex, striatum, hippocampus, and substantia nigra. In addition, we examined whether a concomitant treatment with lipoate, a dithiol that may act as a target of S-carbamoylation, can prevent these changes. Cyanate-inhibited sulfurtransferase activities and lowered sulfide level, which was accompanied by a decrease in glutathione concentration and elevation of reactive oxygen species level in almost all rat brain structures. Lipoate administered in combination with cyanate was able to prevent the above-mentioned negative cyanate-induced changes in a majority of the examined brain structures. These observations can be promising for chronic renal failure patients since lipoate can play a double role in these patients contributing to efficient antioxidant defense and protection against cyanate and cyanide toxicity.

## Introduction

In biological systems, cyanate shows the highest reactivity with sulfhydryl (SH) groups of proteins (Arlandson et al. 2001; Wisnewski et al. 1999). Since –SH groups are present in active centers of enzymes participating in anaerobic cysteine transformation, e.g., cystathionase (CSE, EC 4.2.1.15) and mercaptopyruvate sulfurtransferase (MPST, EC 2.8.1.2), and sulfane sulfur transporting enzyme, e.g., rhodanese–thiosulfate sulfurtransferase (TST, EC 2.8.1.1) (Nagahara et al. 1995, 2003), it was hypothesized that cyanate could influence the activity of these enzymes as well as the level of the main cellular antioxidant, glutathione (GSH).

**Fig. 1:**
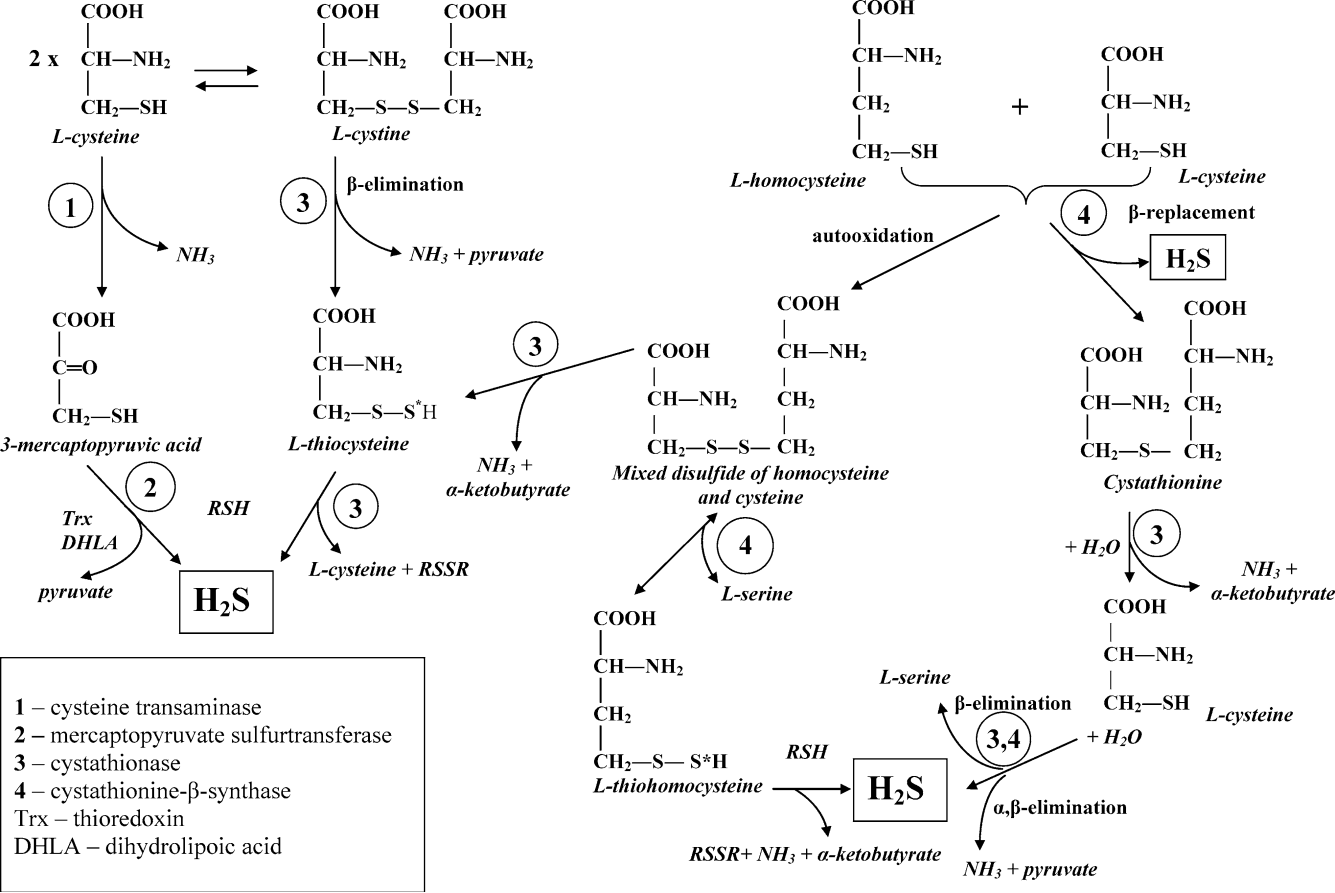
Sulfane sulfur (S*) formation by biodegradation of l-cystine to l-thiocysteine, catalyzed by CSE (3) and of homocysteine and cysteine mixed disulfides to l-thiohomocysteine, catalyzed by CBS (4). The main pathways of hydrogen sulfide (H_2_S) formation: by desulfurization of 3-mercaptopyruvic acid catalyzed by MPST (2); during cystathionine synthesis catalyzed by CBS (4); in β- and α,β-elimination reactions of l-cysteine, catalyzed by CSE (3) and CBS (4); in the reaction of persulfides (e.g., thiocysteine, thiohomocysteine) with an excess of cellular reducers (RSH)

Cysteine, which is formed from exogenous methionine, is both the GSH precursor and the main source of active sulfur in tissues. Sulfur-containing compounds can possess a stably bound sulfur, as that in glutathione and cysteine or labile sulfur, e.g., acid labile, sulfane sulfur (S*), and protein bound sulfane sulfur. S* is a highly reactive sulfur in 0 or −1 oxidation state covalently bound to another sulfur atom. The pool of sulfane sulfur-containing compounds comprises, e.g., polysulfides (R–S–S_n_*–S–R), thiosulfate (S_2_O_3_^2−^), and persulfides (R–S–S*H), which are formed during biodegradation of cystine and mixed disulfides (Fig. 1) in the presence of CSE and cystathionine β-synthase (CBS, EC 4.2.1.22). S* plays an important role in cyanide (CN^−^) to thiocyanate (SCN^−^) detoxification catalyzed by TST, MPST, and by CSE (Nagahara et al. 1995, 1999, 2003; Toohey 1989). Most of the labile sulfur is liberated as inorganic sulfides, e.g., H_2_S, HS^−^, or S^2−^, in the presence of acids or reducing agents (Toohey 1989, 2011; Ubuka 2002). On the other hand, hydrogen sulfide can be stored in the form of protein bound sulfane sulfur (Shibuya et al. 2009). It indicates a close relation between H_2_S and sulfane sulfur. In the brain, hydrogen sulfide (H_2_S) fulfills the function of neurotransmitter and vasodilator. It was believed earlier that the formation of the main pool of hydrogen sulfide in the brain was catalyzed by CBS, while in the periphery by cystathionine γ-lyase—CSE (Chen et al. 2004; Li et al. 2006; Stipanuk 2004; Toohey 2011). However, the most recent studies have demonstrated that hydrogen sulfide synthesis in the brain tissue is catalyzed mostly by MPST (in the presence of thioredoxin or dihydrolipoic acid) (Shibuya et al. 2009; Mikami et al. 2011) (Fig. 1).

**Fig. 2:**
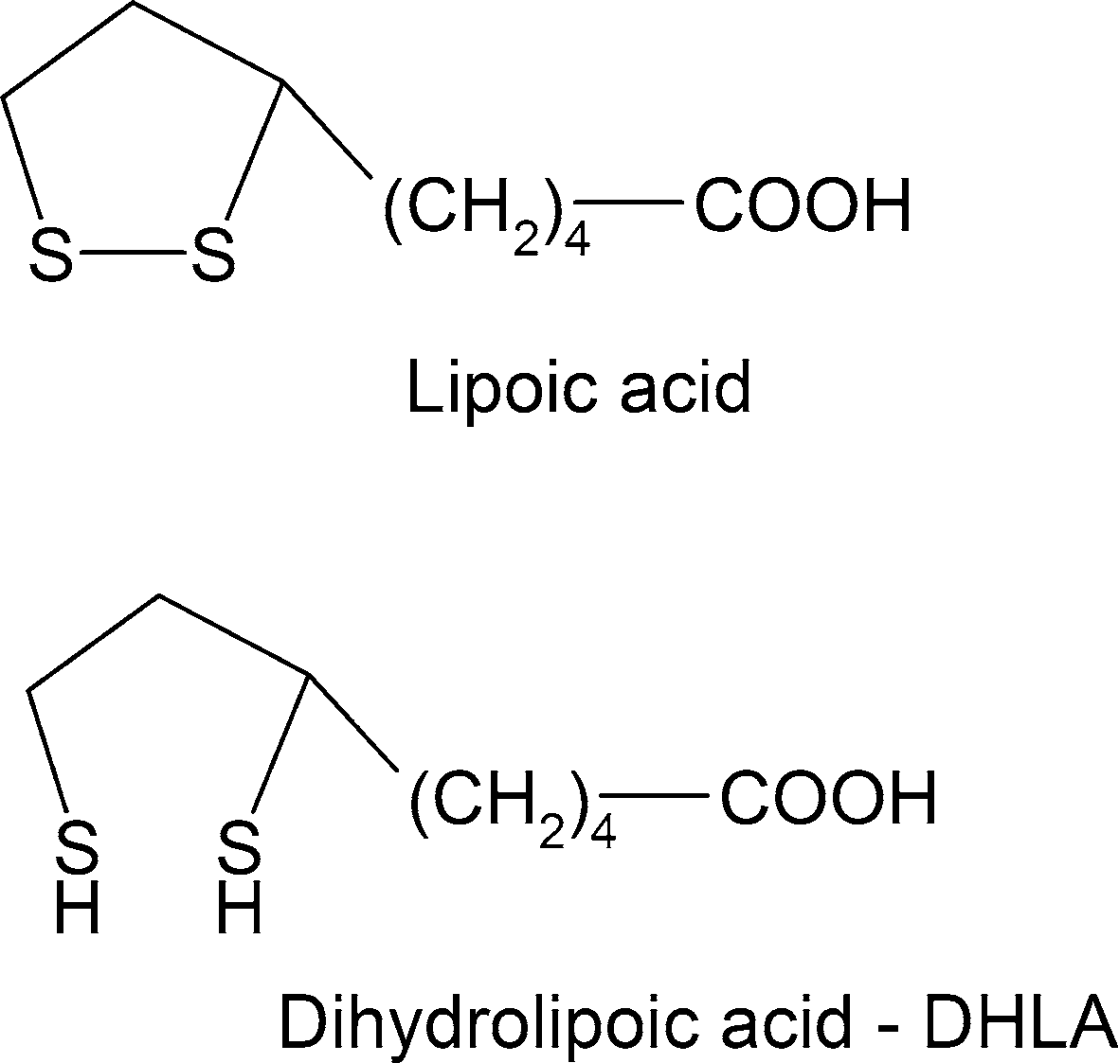
The structure of oxidized (LA) and reduced (DHLA) form of lipoic acid

**Fig. 3:**
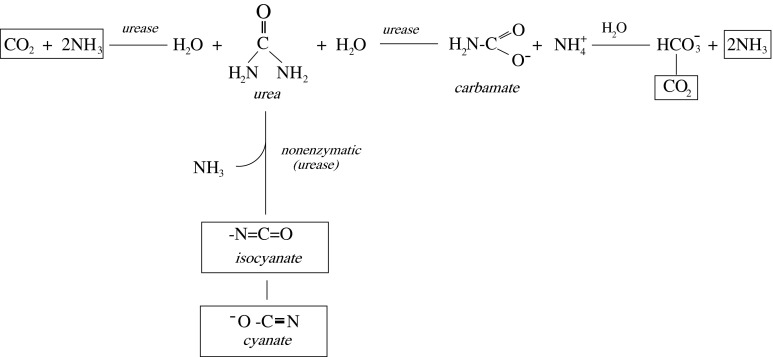
Urea breakdown to ammonia (NH_3_) and carbon dioxide (CO_2_) catalyzed by urease, and to isocyanate (NCO^−)^ and cyanate (OCN^−^) (nonenzymatic and enzymatic elimination)

Our earlier studies demonstrated prooxidative properties of cyanate and its inhibitory action on enzymatic activities of sulfurtransferases while lipoic acid [IUPAC name: 5-(1,2-dithiolane-3-yl)pentanoic acid] (Fig. 2) prevented that effect in the rat liver (Sokolowska et al. 2011). Hence, we expected to see similar effect in the rat brain. However, the effect of cyanate on antioxidant enzyme activity and H_2_S level was unknown. Since oxidative stress can differently affect antioxidant enzyme activity in various brain regions (Severynovs’ka et al. 2006; Mladenović et al. 2012), we expected to observe diverse effects of cyanate in different brain structures. The effect of cyanate on the organism can be particularly significant in uremia in which plasma level of urea (a cyanate (OCN^−^) and isocyanate (NCO^−^) precursor (Fig. 3) is significantly increased (Beddie et al. 2005; Estiu and Merz 2007; Vanholder et al. 2003). This compound can also affect brain tissue because both, in vitro and in vivo studies with ^14^C radiolabeled cyanate documented its incorporation into cerebral proteins in the process of S- and N-carbamoylation (Crist et al. 1973; Fando and Grisolia 1974).

Lipoic acid (LA) (Fig. 2), due to its structure, may act as a target of carbamoylation and in this way may protect –SH groups of proteins. In addition, LA is a very strong antioxidant able to quench free radicals and restore the activity of other antioxidants. Since LA participates also in the regulation of sulfane sulfur metabolism, it is probable that it can show a protective action against harmful effects of cyanate in brain structures (Bilska et al. 2008; Smith et al. 2004). These data prompted us to investigate the effect of cyanate and lipoate alone and in combination on anaerobic cysteine transformation, in particular on hydrogen sulfide level and activity of sulfane sulfur synthetic and transport enzymes, and on the concentrations of pro- and antioxidants in different structures of the brain: cortex, striatum, hippocampus, and substantia nigra (SN).

## Materials and Methods

### Animals

The experiments were carried out on male Wistar rats weighing ~250 g. The animals were kept under standard laboratory conditions and were fed a standard diet. All experiments were carried out in accordance with the National Institutes of Health Guide for the Care and Use of Laboratory Animals and with approval of the Bioethics Commission as compliant with the Polish Law (21 August 1997) (permission no. 645, 23.04. 2009). Animals were assigned to four groups, containing 7 animals each. Groups were treated as follows.

Group 1 

Group 2 

Group 3 

Group 4 

Cyanate (KCNO) was administered intraperitoneally (i.p.) at a dose of 200 mg/kg b.w. The dose of cyanate was chosen based on the data presented in the article by Tor-Agbidye et al. (1999). The dose of lipoic acid (LA) 50 mg/kg b.w. i.p. twice was chosen on the basis of our earlier studies (Bilska et al. 2008). Efficacy of this dose was confirmed by literature data (Micili et al. 2013; Shay et al. 2009).

Animals were sacrificed 2.5 h after the first injection, because there had to be an interval between preventive lipoate administration and cyanate dose, and then the next therapeutic LA treatment (aimed to lower cyanate action). Subsequently, the above-mentioned brain structures were isolated and stored at −80 °C until further experiments were performed.

### Biological Material

The tissues were weighed and homogenized in 400 μl of an ice-cold phosphate buffer, pH 7.4 per 100 mg of the tissue. Substantia nigra specimens (left and right ca. 5–8 mg) originating from three rats were pooled and homogenized in the same manner (on the average 80 μl of an ice-cold phosphate buffer, pH 7.4 per 20 mg of the tissue).

### Chemicals

Potassium cyanide (KCN), dithiothreitol, *p*-phenylenediamine, *N*-ethylmaleimide (NEM), β-nicotinamide adenine dinucleotide reduced form (NADH), 5,5′-dithio-bis-2-nitrobenzoic acid (DTNB), NADPH, mercaptopyruvic acid sodium salt l-homoserine, pyridoxal 5′-phosphate monohydrate, 3-methyl-2-benzothiazolinone hydrazone hydrochloride monohydrate, and lactic dehydrogenase (LDH), potassium cyanate (KNCO), glutathione reduced form, glutathione reductase, and α-lipoic acid sodium salt were provided by Sigma Chemical Co. (St. Louis, MO, USA). Formaldehyde, ferric chloride (FeCl_3_), thiosulfate, and all the other reagents were obtained from the Polish Chemical Reagent Company (P.O.Ch, Gliwice, Poland).

## Methods

1. Glutathione (GSH) level was determined by the method of Ellman (1959).
2. γ-Glutamyltransferase (γGT) activity was assayed by the method of Orłowski and Meister (1963).
3. Activity of glutathione peroxidase (GP*x*) was assayed by the method of Flohe and Gunzler (1984).
4. Catalase activity was determined according to Aebi (1984).
5. Activity of rhodanese (TST) was determined using the method of Sörbo (1955).
6. MPST activity was assayed according to Valentine and Frankelfeld (1974).
7. CSE activity was determined by the method of Matsuo and Greenberg (1958).
8. Sulfane sulfur (S*) level was assayed by a cold cyanolysis method (Wood 1987).
9. Hydrogen sulfide/sulfides level was determined using a modification of the method of Tamizhselvi et al. (2007).

Briefly, the assay was based on the fact that H_2_S produced in the brain tissues reacts with Zn(CH_3_COO)_2_ to form ZnS which than reacts with *p*-phenylenediamine yielding a fluorescent dye (thionein) in the presence of ferric chloride (FeCl_3_). Aliquots of homogenate (125 μl) were mixed with 1 mM Na_2_CO_3_ (125 μl) and 5 % zinc acetate (125 μl) followed by incubation at room temperature (30 min). Next, *p*-phenylenediamine (12.5 mM), 40 mM FeCl_3_ in 6 M HCl, and H_2_O (125 μl) were added. After a 10-min development at room temperature, the samples were centrifuged for 5 min in 10,000×*g* and fluorescence of the mixture was measured (Ex 600 nm, Em 623 nm). H_2_S concentrations were read from a calibration curve prepared from thionein (2.5–25 μM).

10. Reactive oxygen species (ROS) level was assayed by the method of Bondy and Guo (1994).
11. Protein content was determined according to Lowry et al. (1951).

### Statistical Analysis

The results are presented as the mean ± SEM for each group. Statistically significant differences between groups were calculated using a two-way ANOVA, followed (if significant) by the Tukey test for comparison between the examined groups.

## Results

### GSH Concentration

**Fig. 4:**
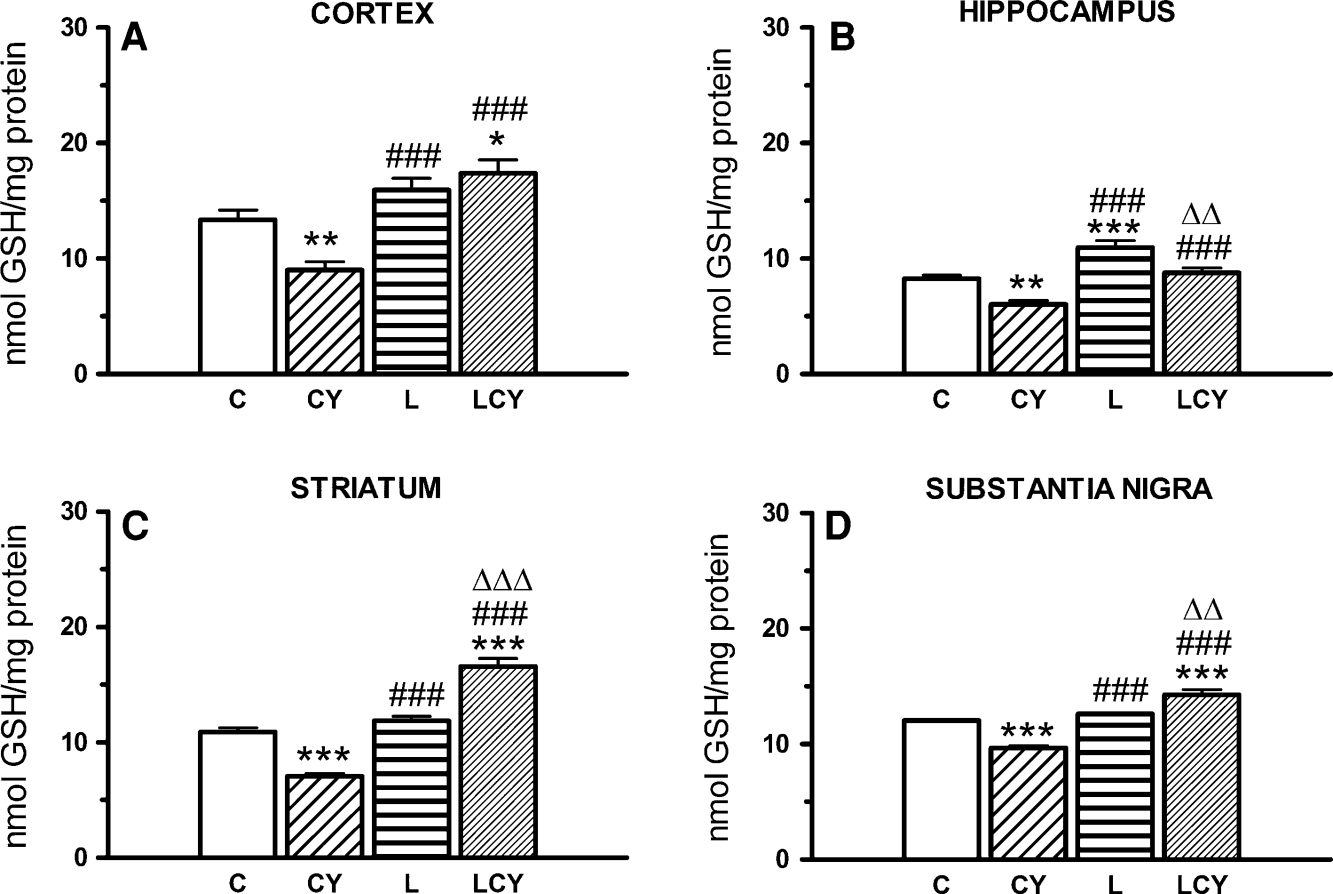
The effect of acute administration of cyanate (CY, 200 mg/kg) and lipoate (L, 50 mg/kg twice), alone and in combination (LCY) on the total GSH level. Data are presented as the mean ± SEM, *n* = 21 for each group of cortex samples, *n* = 10 for hippocampus samples, *n* = 9 for each group of striatum samples, and *n* = 5 for substantia nigra samples. *Symbols* indicate significance of differences in the Tukey test, ***P < 0.001, **P < 0.01, *P < 0.05 versus control (*C*); ^###^ *P* < 0.001 versus CY group; ^∆∆∆^ *P* < 0.001, ^ΔΔ^ *P* < 0.01 versus L-treated group

Cyanate significantly lowered GSH concentration in all structures of the rat brain under study (Fig. 4).

The most pronounced decrease in GSH content after cyanate injection was observed in the cortex and in the striatum (by 33 and 35 % vs. control, respectively), moderate in the hippocampus (by 27 % of the control), and the smallest one in the substantia nigra (by 20 %). Lipoate alone treatment significantly elevated GSH level only in the hippocampus (by 32 % of the control group). Lipoate administrated jointly with cyanate (before and after cyanate) enhanced GSH concentration the most distinctly in the cortex and striatum (by 30 and 52 % of the control level, as well as by 93 and 134 % of the cyanate-treated group, respectively) but slightly weaker in the hippocampus and substantia nigra (SN) (by 18 and 6 % vs. control group, and by 45 and 47 % of the cyanate-treated group, respectively) (Fig. 4).

### γGT Activity

**Fig. 5:**
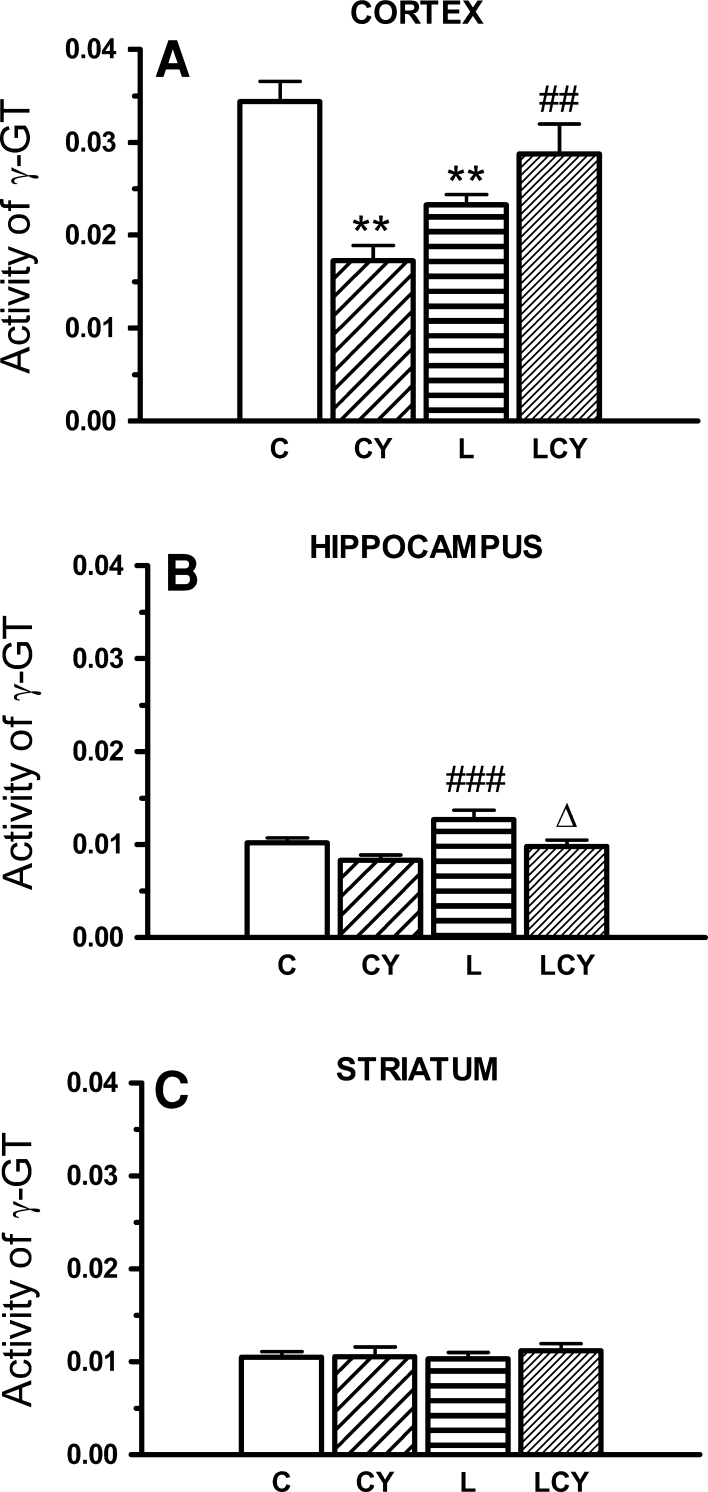
The effect of acute administration of cyanate (CY, 200 mg/kg) and lipoate (L, 50 mg/kg twice), alone and in combination (LCY) on the enzymatic activity of γ-glutamyl transferase (γ-GT) expressed in U/mg (μmol of *p*-nitroaniline formed during 1 min/mg protein). Data are presented as the mean ± SEM, *n* = 18 for each group of cortex samples, *n* = 12 for hippocampus samples, and *n* = 9 for each group of striatum samples. *Symbols* indicate significance of differences in the Tukey test, **P < 0.001 versus *C*; ^###^ *P* < 0.001, ^##^ *P* < 0.01 versus CY; ^∆^ *P* < 0.05 versus L-treated group

Cyanate administration significantly reduced γ-glutamyltransferase (γGT) activity, but only in the cortex (by 50 % of the control group). Lipoate treatment decreased activity of the enzyme versus control (by 32 %), but joint treatment with cyanate and lipoate partially restored activity of the enzyme in this structure (by 66 % of cyanate-treated group), however, it did not reach activity of this enzyme in the control (Fig. 5).

### GP*x* and Catalase Activities

**Fig. 6:**
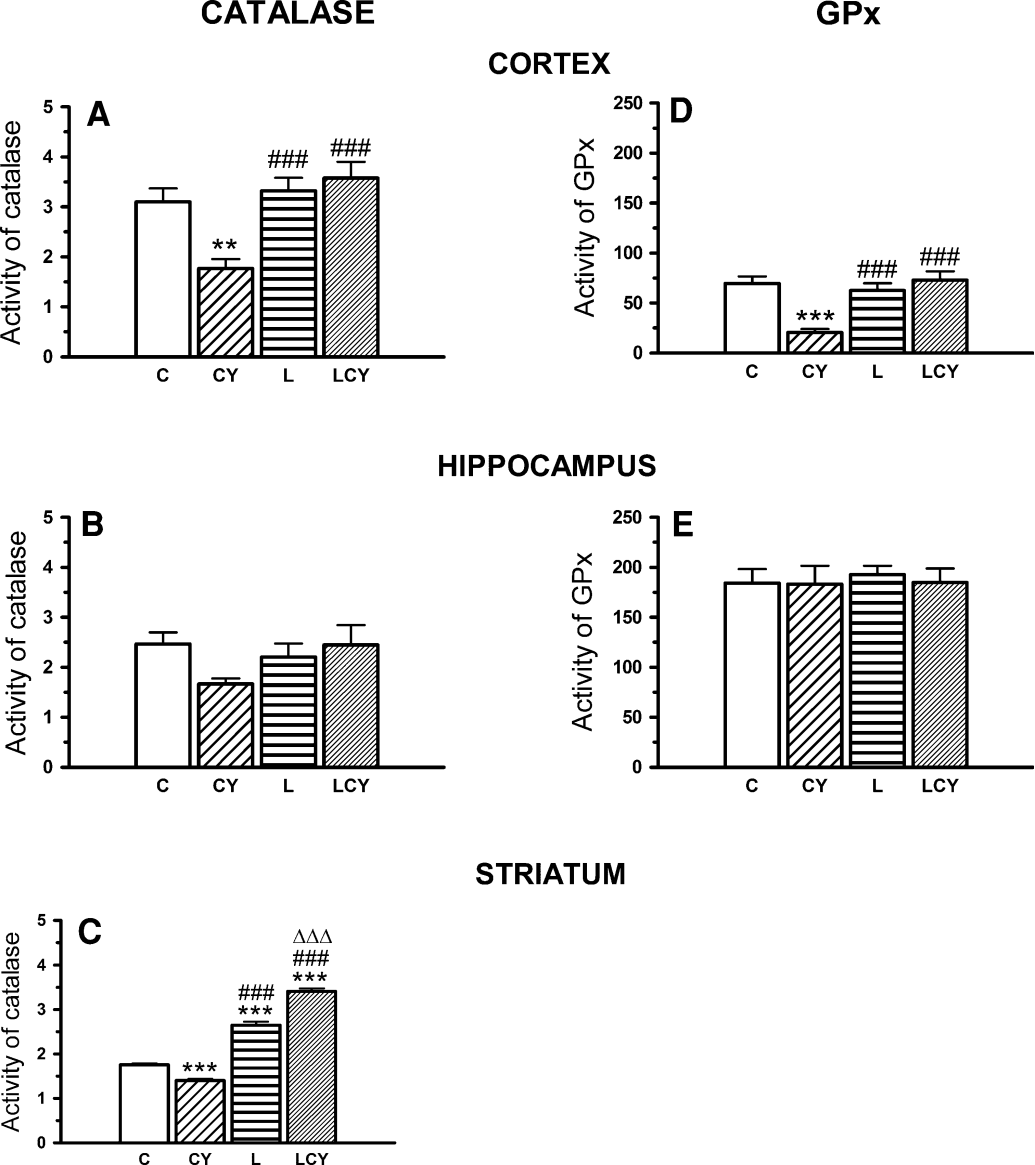
The effect of acute administration of cyanate (CY, 200 mg/kg.) and lipoate (L, 50 mg/kg twice), alone and in combination (LCY) on the enzymatic activities of catalase (**a**–**c**) and glutathione peroxidase (GP*x*; **d**, **e**). Activity of catalase was expressed in U/mg (μmol of H_2_O_2_ degraded by the enzyme/mg of protein/min), while activity of glutathione peroxidase was expressed in mU/mg (nmol of GSH, oxidized by the enzyme during 1 min/mg protein). Data are presented as the mean ± SEM, *n* = 21 for each group of cortex samples, *n* = 10 for hippocampus samples, and *n* = 9 for each group of striatum samples. *Symbols* indicate significance of differences in the Tukey test, ***P < 0.001, **P < 0.05 versus control (*C*); ^###^ *P* < 0.001 versus CY; ^∆∆∆^ *P* < 0.001 versus L-treated group

Cyanate significantly lowered GP*x* activity in the cortex (by 70 % of the control) and catalase activity both in the cortex and striatum (by 43 and 20 % of the control, respectively) (Fig. 6). Lipoate alone maintained GP*x* activity at the control level, while administrated in combination with cyanate restored activity of this enzyme versus control group and simultaneously raised it by 250 % versus the cyanate group. Lipoate alone maintained catalase activity in the cortex at the control level, while in the striatum increased it markedly in comparison to the control group (by 50 %). Lipoate administrated jointly with cyanate not only restored the catalase activity in the cortex and striatum, but even enhanced it markedly in the striatum (by 93 % of the control). Consequently in the cortex and striatum, catalase activity was significantly higher (by 102 and 142 %, respectively) than in the cyanate group (Fig. 6).

### TST and MPST Activity

Administration of cyanate markedly reduced TST activity in the substantia nigra (SN) (by 32 % of the control) and in the cortex (by 31 %), but slightly more weakly in the striatum (by 20 %). MPST activity in the SN (by 25 % of the control) and striatum (by 21 %) also declined. Cyanate did not change activities of both enzymes in the hippocampus. Lipoate alone enhanced significantly activities of these enzymes in the hippocampus; TST by 32 % of the control and by 66 % of the cyanate-treated group, while MPST by 38 % of the control and by 58 % of the cyanate-treated group, and TST activity in the SN (by 6 % of the control and 57 % of the cyanate-treated group). Lipoate administrated jointly with cyanate significantly raised TST activity versus control only in the hippocampus (by 30 %); while in the cortex (by 47 %), SN (44 %) and in the striatum (by 17 %) when compared to the cyanate-treated group.

**Fig. 7:**
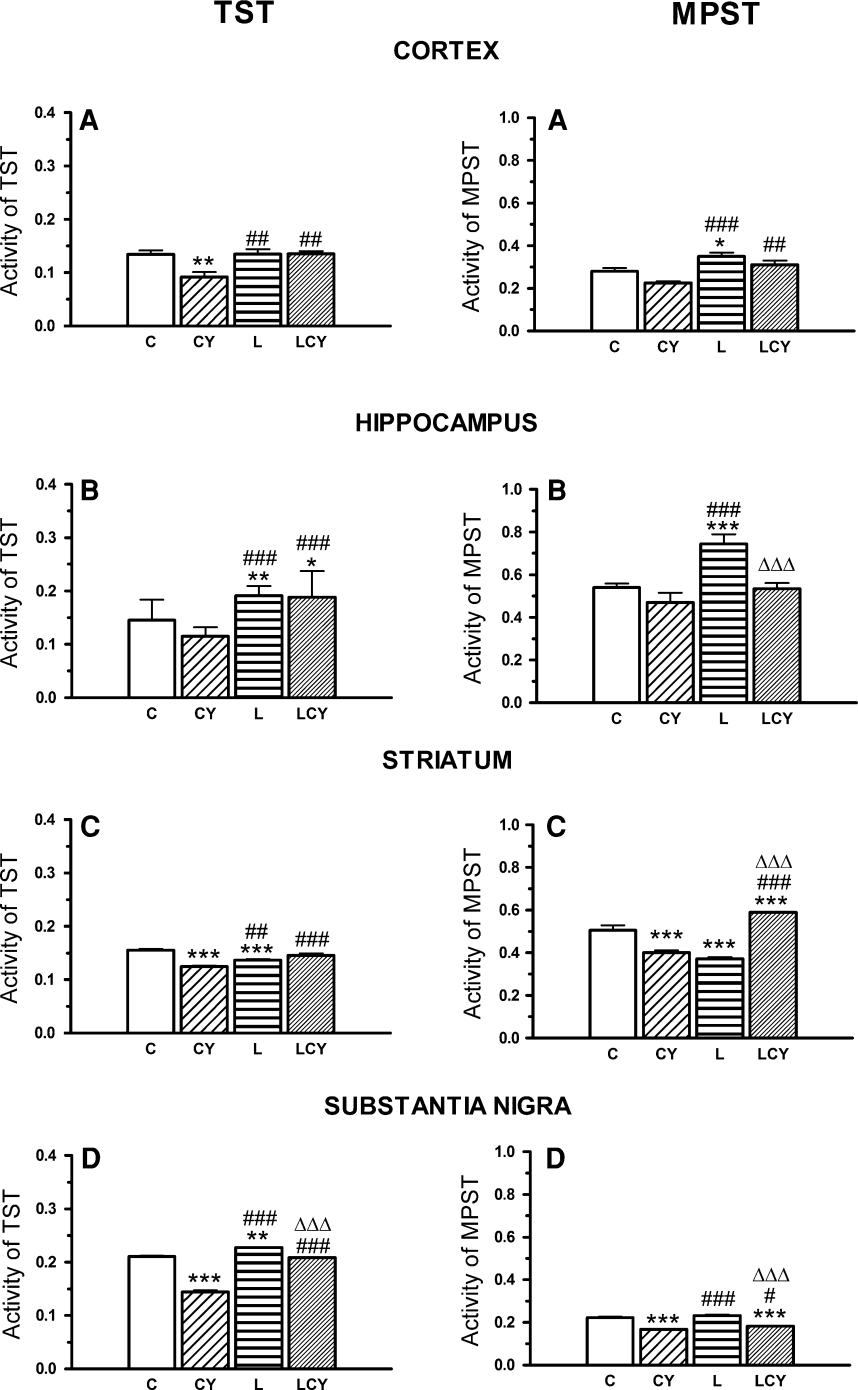
The effect of acute administration of cyanate (CY, 200 mg/kg) and lipoate (L, 50 m/kg twice), alone and in combination (LCY) on enzymatic activities of thiosulfate (TST) and mercaptopyruvate (MPST) sulfurtransferases (**a**–**d**) in the rat brain structures. TST and MPST activities were expressed in U/mg (μmoles of SCN^−^/mg of protein/min; μmoles of pyruvate/mg of protein/min, respectively). Data of TST and MPST activities are presented as the mean ± SEM, *n* = 18, *n* = 12, *n* = 9, and *n* = 6 for each group of cortex, hippocampus, striatum, and substantia nigra samples, respectively. *Symbols* indicate significance of differences in the Tukey test, ***P < 0.001, **P < 0.01, *P < 0.05 versus control (*C*); ^###^ *P* < 0.001, ^##^ *P* < 0.01, ^#^ *P* < 0.05 versus CY; ^ΔΔΔ^ *P* < 0.001 versus L-treated group

As to MPST, apart from the hippocampus, lipoate alone enhanced activity of the enzyme in the cortex (by 25 % of the control and by 56 % of the cyanate-treated group), while decreased it in the striatum (by 26 % of the control). After the combined treatment with cyanate and lipoate, MPST activity was increased in the striatum (by 17 %, when compared to the control and by 47 % vs. the cyanate-treated group), in the cortex and in the SN (by 38 % and by 10 % of the cyanate-treated group, respectively). Lipoate in combination with cyanate did not changed MPST activity in the hippocampus (Fig. 7).

#### Cystathionase (CSE) Activity

**Fig. 8:**
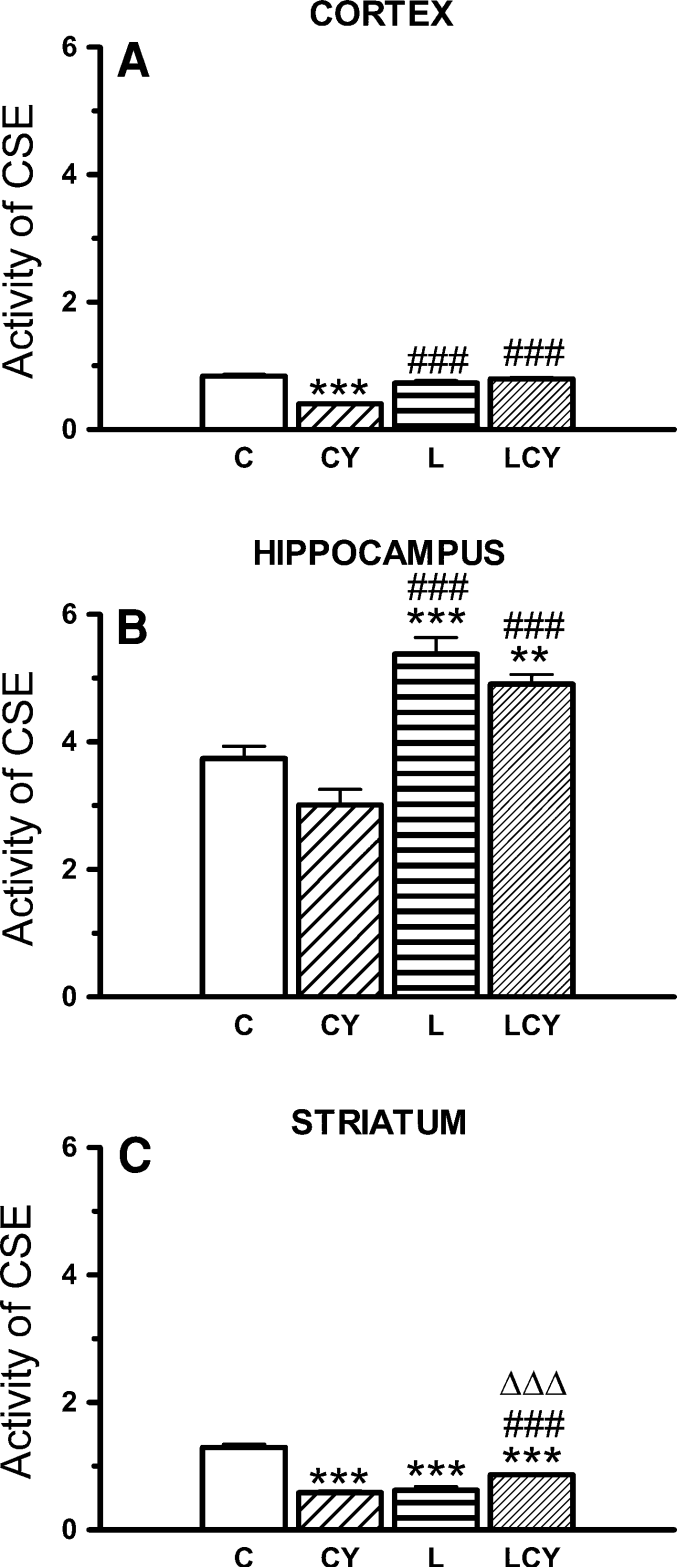
The effect of acute administration of cyanate (CY, 200 mg/kg) and lipoate (L, 50 mg/kg twice), alone and in combination (LCY) on the enzymatic activity of γ-cystathionase (CSE; **a**–**c**). CSE activity was expressed in U/mg (μmoles of α-ketobutyric acid formed from homoserine/mg of protein/min). Data are presented as the mean ± SEM, *n* = 18 for each group of cortex samples, *n* = 9 for hippocampus and striatum samples. *Symbols* indicate significance of differences in LSD test, ***P < 0.001, **P < 0.01, *P < 0.05 versus control (*C*); ^###^ *P* < 0.001) versus CY; ^ΔΔΔ^ *P* < 0.001 versus L-treated group

CSE activity was the highest in the hippocampus. Cyanate significantly lowered CSE activity both in the cortex and striatum (by 52 % of the control). Lipoate alone enhanced activity of the enzyme versus control only in the hippocampus (by 44 %). Lipoate administrated jointly with cyanate (before and after cyanate) elevated its activity versus cyanate group, in all structures under study, i.e., in the cortex (by 98 % of the cyanate-treated group), in the striatum (by 39 %), and hippocampus (by 65 % of the value after cyanate treatment) (Fig. 8).

#### Sulfide and Sulfane Sulfur Level

**Fig. 9:**
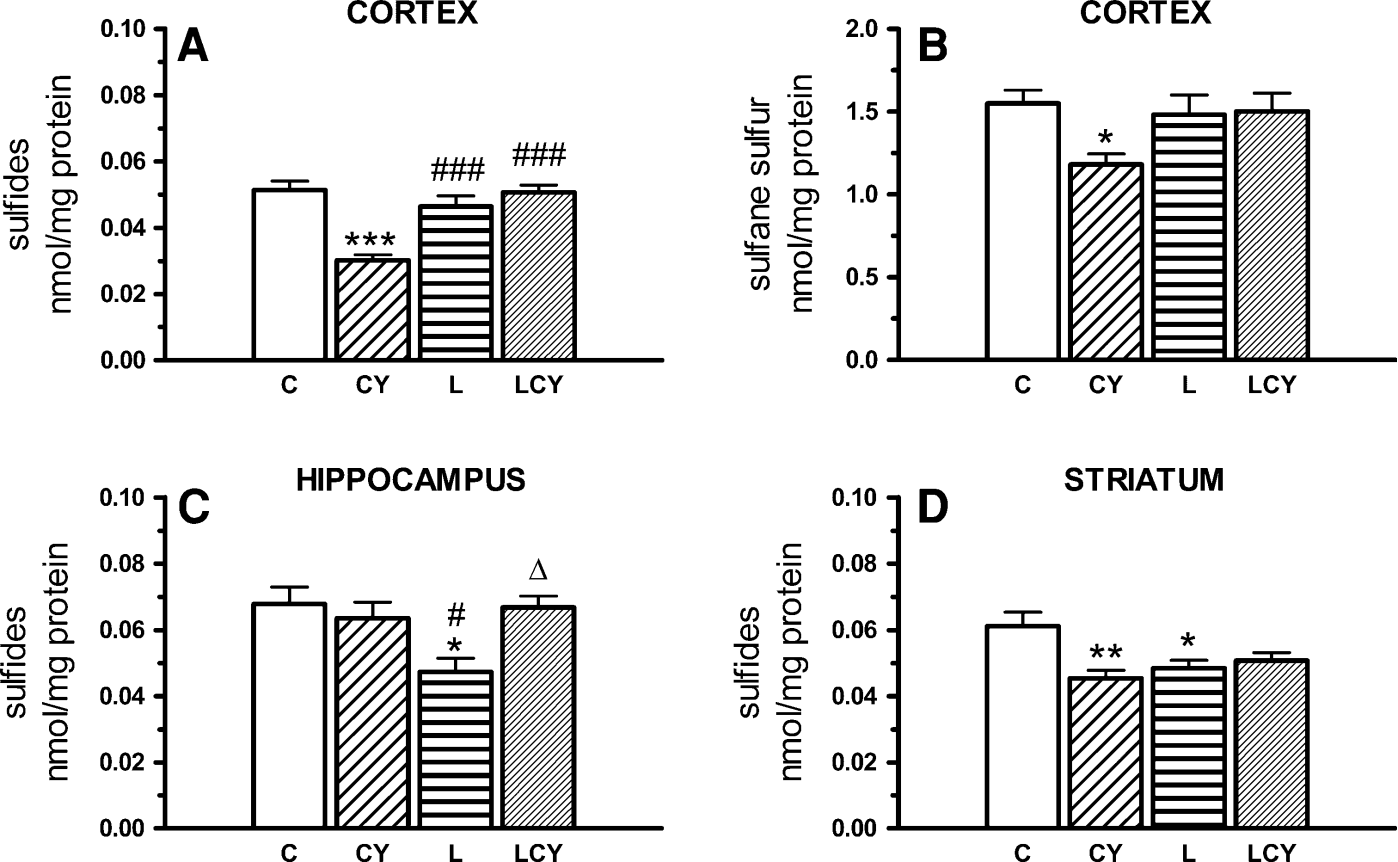
The effect of acute administration of cyanate (CY, 200 mg/kg.) and lipoate (L, 50 mg/kg twice), alone and in combination (LCY) on the level of sulfides (**a**, **c**, **d**) and sulfane sulfur (S*) (**b**). Concentration of sulfides was expressed in nmoles of thionine/mg of protein and of sulfane sulfur in nmoles of SCN^−^/mg. Data are presented as the mean ± SEM, *n* = 18 for each group of cortex samples, n = 12 for hippocampus and n = 9 for each group of striatum samples. Symbols indicate significance of differences in the Tukey test, ***P < 0.001, **P < 0.01, *P < 0.05 versus control (*C*); ^###^ *P* < 0.001, ^#^ *P* < 0.05 versus CY; ^Δ^ *P* < 0.001 versus L-treated group

Cyanate significantly lowered the level of sulfides/H_2_S in the cortex (by 41 % of the control) and striatum (by 30 % of the control), and sulfane sulfur (S*) level in the cortex (the only structure in which S* was determined because of the lack of material from other structures) (by 24 % of the control). Lipoate alone decreased the concentration of sulfides in the striatum, by 21 % and in the hippocampus, by 30 % of the control. In the cortex, lipoate administrated alone elevated neither the level of S* nor sulfides versus control, but combined treatment with cyanate and lipoate raised the level of sulfides in the cortex (by 67 % of cyanate-treated group) and restored the concentration of sulfides to the control level (Fig. 9).

#### ROS Level

**Fig. 10:**
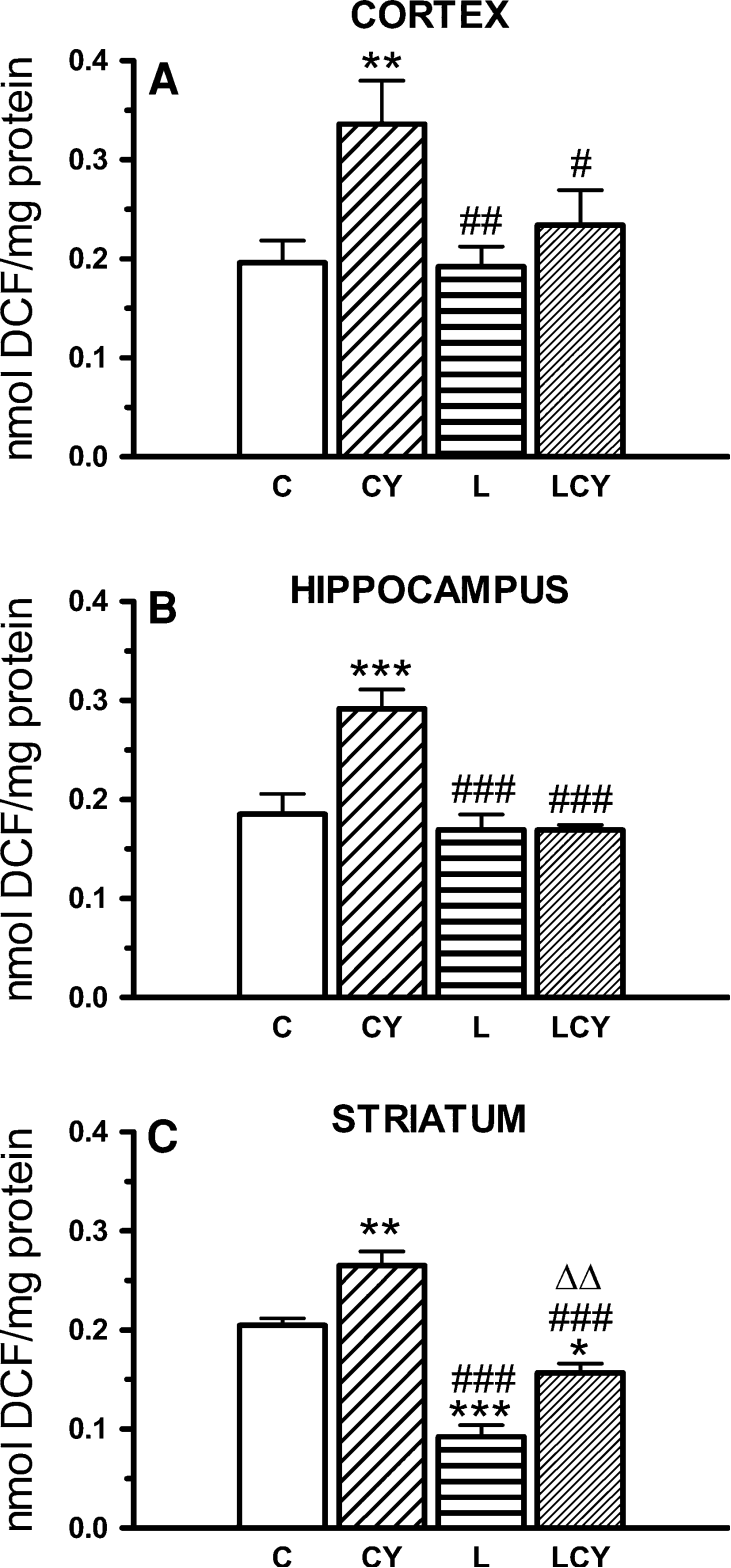
The effect of acute administration of cyanate (CY, 200 mg/kg.) and lipoate (L, 50 mg/kg twice), alone and in combination (LCY) on the level of reactive oxygen species (ROS; **a**–**c**). Concentration of ROS was expressed in nmoles of 2,7-dichlorofluorescein (DCF)/mg of protein. Data are presented as the mean ± SEM, *n* = 18 for each group of cortex samples, *n* = 11 for hippocampus, and *n* = 9 for striatum samples. *Symbols* indicate significance of differences in the Tukey test, ***P < 0.001, **P < 0.01 versus control (*C*); ^###^ *P* < 0.001, ^##^ *P* < 0.01 versus CY; ^#^ *P* < 0.05 versus CY; ^ΔΔ^ *P* < 0.001 versus L-treated group

After cyanate treatment, the level of free radicals increased in all structures under study: in the cortex by 71 %, in the hippocampus by 58 %, and in the striatum by 30 % of the control group. Lipoate alone significantly decreased the concentration of ROS only in the striatum (by 55 % vs. control). Administration of cyanate and lipoate significantly lowered ROS level in all the structures: in the cortex by 28 % of the cyanate-treated group, in the striatum by 41 % while in the hippocampus by 42 % of the cyanate-treated group, i.e., it restored ROS level in the cortex and hippocampus almost to the control level, while in the striatum even decreased it below this level. (Fig. 10).

## Discussion

Reduction of glutathione (GSH) level as well as sulfurtransferase (TST, CSE) and catalase activities (Figs. 4, 6, 7, 8) accompanied by an increase in free radical level (Fig. 10) was observed in almost all rat brain structures after cyanate treatment, through to different degree. It may indicate prooxidative action of cyanate and following carbamoylation of proteins, especially –SH groups of sulfurtransferases and GSH. Cyanate-induced lowering of GSH level of varying magnitude was also reported by Tor-Agbidye et al. (1999) in different mouse brain structures. On the other hand, carbamoylation is corroborated by reports demonstrating cyanate incorporation into cerebral proteins and a direct cyanate reaction with GSH (Crist et al. 1973; Fando and Grisolia 1974; Stark et al. 1960; Wisnewski et al. 1999). The decrease in GSH level, the most notable in the cortex and in the striatum, can also be the result of CSE inhibition in these structures since this enzyme supplies cysteine, a rate-limiting substrate for glutathione synthesis. GSH (γ-glutamylcysteinyl glycine) biosynthesis in brain cells is catalyzed by γ-glutamylcysteine ligase (GCL), which is a rate-limiting enzyme, and glutathione synthetase (GS). On the other hand, GSH is catabolized to glutamate, cysteine, and glycine which can be reused for GSH synthesis catalyzed by the transmembrane enzyme γGT. Therefore, the decrease in γGT activity observed in the cortex can contribute to the drop in GSH level in this structure. Since GSH is the main cellular antioxidant which participates in reactive oxygen species (ROS) scavenging and removal of hydrogen peroxide and organic peroxides, a decrease in its level elevates ROS level. The cyanate-induced increase in ROS level could also be attributed to the decreased level of hydrogen sulfide in the cortex and in the striatum, because H_2_S inhibits ROS formation under natural conditions by inhibiting NADH oxidase activity (Samhan-Arias et al. 2009). Since physiological concentration of H_2_S also increases the activity of the cystine–glutamate antiporter (Xc^−^) and of γ-glutamylcysteine synthase, the drop in H_2_S concentration in the cortex and in the striatum (Fig. 9) can indirectly affect GSH level (Kimura and Kimura 2004; Kimura et al. 2010). According to Chen et al. (2004), CBS is the only enzyme able to produce H_2_S in the brain. However, biochemical studies of Linden et al. (2008) evidenced CSE expression and H_2_S production in the mouse brain. CSE activity was also present in various regions of the rat brain from a 6-month-old infant at autopsy (Stipanuk 2004). In the hippocampus neither MPST (the main H_2_S-producing enzyme) nor CSE activity was changed by cyanate, which was manifested as an unaltered sulfide/H_2_S level (Figs. 7, 8, 9). The cyanate-induced lowering of H_2_S level in the cortex and striatum correlates with the decrease in CSE activity in both structures and MPST activity in the striatum. The inhibitory effect of cyanate on CSE activity also leads to depletion of the so-called sulfane sulfur pool in the cortex. In addition, the decline in TST and MPST activities (which under physiological conditions are about 5–10 times lower in the brain than in other organs, like the kidney or heart) can increase sensitivity of the brain structures (especially striatum and SN) to the CN^−^ toxicity, leading to the inhibition of cytochrome oxidase, and thus to the decrease in ATP level and neuropathy (de Sousa et al. 2007; Hasuike et al. 2004; Nagahara et al. 1999).

H_2_S was shown to increase the activity of neuronal *N*-methyl-d-aspartate (NMDA) receptor, activating calcium channels and leading to a prolonged enhancement of long-term potentiation (LPT) which is of key significance for some forms of learning and memory (Nagai et al. 2004; Kimura and Kimura 2004). No changes in the hippocampal MPST activity and H_2_S level after acute cyanate treatment can indicate undisturbed perceptive processes in rats under these conditions. On the other hand, hippocampal hydrogen sulfide level, unaffected by cyanate, suggests an undisturbed functioning of the Xc^−^ antiporter to supply cystine, and unhindered GSH synthesis in this structure of the brain (Kimura et al. 2010). In that case, the decrease in GSH level, slightly smaller than in other structures (Fig. 9), could be an immediate consequence of its direct carbamoylation by cyanate.

Although the mechanism of catalase (in the cortex and striatum) and GP*x* (in the cortex) inhibition is not known in detail, there are reports demonstrating a decrease in activities of these enzymes in the rat brain during oxidative stress (Hfaiedh et al. 2012; Bild et al. 2012), and various stress effects on antioxidant enzymes in different brain structures (Mladenovic et al. 2012). However, it would be difficult to compare those results with the present data due to completely different stress conditions.

The increase in GSH level after the combined administration of cyanate and lipoate was the greatest in those structures where it was decreased the most by cyanate, i.e., in the cortex and in the striatum, which can indicate a stimulating lipoate effect during cyanate-induced oxidative stress. Both LA and its reduced form DHLA easily cross the blood–brain barrier thus creating redox system with a low redox potential (*E*_o_ = −0.29 V), capable of regeneration of other cellular antioxidants, including GSH and cysteine (CSH) (by the reduction of respective disulfides: GSSG and CSSC) (Biewenga et al. 1997; Shay et al. 2009). It was confirmed by literature reports indicating the increase in GSH concentration in cell cultures and animal tissues after lipoate administration (Busse et al. 1992; Shay et al. 2009; Wessner et al. 2006). CSE activation after lipoate + cyanate administration, observed in the cortex, striatum, and to the greatest degree in the hippocampus, could lead to elevation of the concentration of cysteine, a GSH precursor (Fig. 1). According to Smith et al. (2004), there are two paths by which LA/DHLA can increase GSH level: (1) boosting of cysteine availability by extracellular cystine reduction and (2) facilitation of cystine uptake by enhancement of Xc^−^ transport system (cystine/glutamate antiporter) expression. The recent hypothesis suggests that LA increases total antioxidant capacity of cells by induction of antioxidant response element (ARE)-regulated gene transcription, including those encoding γ-glutamylcysteine ligase, an enzyme participating in the first stage of GSH synthesis. ARE region can also partially regulate of Xc^−^ system contributing to transport of cystine, which is the main substrate for GSH production (Shay et al. 2009).

**Fig. 11:**
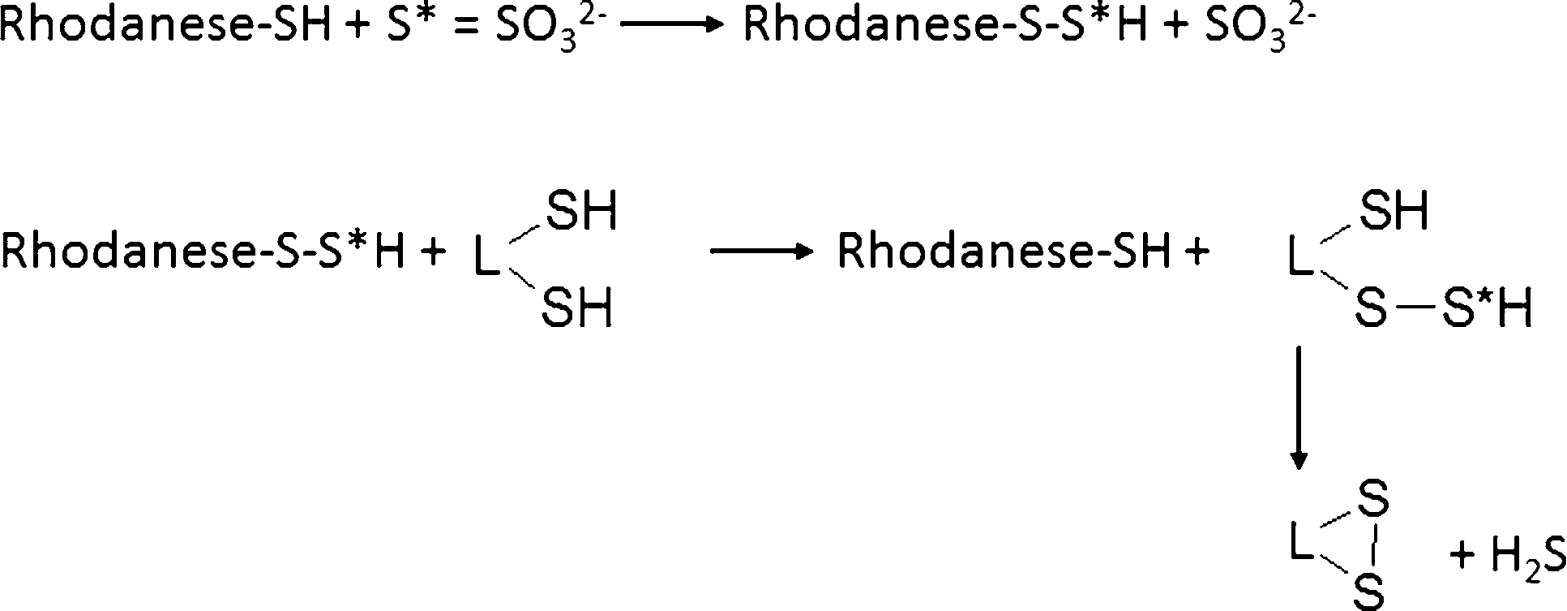
Reactivation of rhodanese in the reaction with DHLA, leading to H_2_S production. The persulfide form of rhodanese (rhodanese–S–S*H) is inactive contrary to the hydrosulfide form (rhodanese–S–H)

Lipoate was also able to restore sulfutransferase activities (Figs. 7, 8) in all structures under study (except for MPST activity in the hippocampus which was not changed by cyanate). Lipoate’s ability to restitute activity of rhodanese (and probably other sulfurtransferases possessing S* binding domain, e.g., MPST and CSE) (Toohey 2011) can be attributed to the fact that the reduced form of lipoate can be an acceptor of sulfane sulfur from the rhodanese active center (Villarejo and Westley 1963) (Fig. 11). It is accompanied by H_2_S formation, confirmed by its increased cortical level after lipoate administration (Fig. 9), which could lead to γ-glutamylcysteine synthetase and cystine transporter (Xc^−^) activation and acceleration of GSH synthesis (Kimura and Kimura 2004). Since hydrogen sulfide more increases cysteine than cystine transport, and parallely cysteine uptake by ASC transporter is faster than cystine uptake by Xc^−^ system, it appears that ASC-involving mechanism can be decisive for elevation of GSH synthesis by H_2_S (Kimura et al. 2010; Smith et al. 2004). To sum up three factors could contribute to the reduction of ROS level after the combined administration of cyanate and lipoate: (1) the increase in GSH level (in all studied structures), (2) the increase in the activity of antioxidant enzymes: catalase (in the cortex and striatum) and GP*x* (in the cortex), and (3) the increase in H_2_S concentration (in the cortex), which inhibits ROS formation (Samhan-Arias et al. 2009). All these factors may be decisive for antioxidant protection of neurons in the cortex and striatum. Additional protection can be obtained due to LA-induced reestablishment of the activities of sulfurtransferases, involved in the production of strongly antioxidant sulfane sulfur. Thus, lipoate administered in combination with cyanate was able to restore cyanide detoxifying capabilities to brain cells. The protective effect of LA administrated in a rat sciatic nerve injury model is another confirmation of restorative lipoate action on the nervous tissue (Ranieri et al. 2010). The increased level of oxidized and carbamoylated low density lipoproteins (LDL) and proteins, detected in atherosclerotic plaques and cholesterol deposits, can suggest a role of both cyanate and oxidative stress in pathogenesis of atherosclerosis and cerebral stroke (Asci et al. 2008; Shah et al. 2008; Wang et al. 2007). Since LA decreased ROS level (Fig. 10) and concomitantly might act as a target for carbamoylation, thus lowering cLDL level, it is able both to alleviate the symptoms of neuropathy observed in CRF patients and to decrease the risk of cerebral stroke. Although cyanate toxicity after acute treatment is relatively low, long-term effects of its action can be much more serious (Alter et al. 1974).

## Conclusions

This article presents the studies demonstrating that the treatment of rats with cyanate alone lowered GSH level and raised reactive oxygen species (ROS) level in all structures of the brain. Besides, cyanate was shown to inhibit TST and CSE activities, to decrease sulfide/hydrogen sulfide level in all structures except for the hippocampus, and to inhibit the activities of MPST (in the striatum and substantia nigra), catalase (in the striatum and cortex), and peroxidase (in the cortex). This indicates that cyanate inhibits anaerobic cysteine transformation and shows prooxidant action in almost all structures of the brain. From among the above-mentioned changes, lipoate administered in combination with cyanate was able to correct ROS and GSH levels, as well as activities of sulfurtransferases, catalase, and peroxidase. It indicates that lipoate can prevent prooxidant cyanate action and cyanate-induced inhibition of enzymes engaged in anaerobic cysteine transformation and cyanide detoxication as well as can activate antioxidant enzymes. These observations can be promising for prophylaxis and alleviation of symptoms of cerebral stroke and neurodegenerative diseases especially in chronic renal failure patients since lipoate can play a dual role in these patients contributing to efficient antioxidant defense and protection against cyanate and cyanide toxicity.
